# Cross Talk with Hematopoietic Cells Regulates the Endothelial Progenitor Cell Differentiation of CD34 Positive Cells

**DOI:** 10.1371/journal.pone.0106310

**Published:** 2014-08-28

**Authors:** Sang-Mo Kwon, Jun-Hee Lee, Sang-Hun Lee, Seok-Yun Jung, Da-Yeon Kim, Song-Hwa Kang, So-Young Yoo, Jong-Kyu Hong, Ji-Hye Park, Jung-Hee Kim, Sung-Wook Kim, Yeon-Ju Kim, Sun-Jin Lee, Hwi-Gon Kim, Takayuki Asahara

**Affiliations:** 1 Laboratory for Vascular Medicine and Stem Cell Biology, Medical Research Institute, Department of Physiology, School of Medicine, Pusan National University, Yangsan, Korea; 2 Soonchunhyang Medical Science Research Institute, Soonchunhyang University Seoul Hospital, Seoul, Korea; 3 Department of Obstetrics and Gynecology, School of Medicine, Pusan National University, Yangsan, Korea; 4 Department Regenerative Medicine Science, Tokai University School of Medicine, Isehara, Japan; French Blood Institute, France

## Abstract

**Introduction:**

Despite the crucial role of endothelial progenitor cells (EPCs) in vascular regeneration, the specific interactions between EPCs and hematopoietic cells remain unclear.

**Methods:**

In EPC colony forming assays, we first demonstrated that the formation of EPC colonies was drastically increased in the coculture of CD34^+^ and CD34^−^ cells, and determined the optimal concentrations of CD34^+^ cells and CD34^−^ cells for spindle-shaped EPC differentiation.

**Results:**

Functionally, the coculture of CD34^+^ and CD34^−^ cells resulted in a significant enhancement of adhesion, tube formation, and migration capacity compared with culture of CD34^+^ cells alone. Furthermore, blood flow recovery and capillary formation were remarkably increased by the coculture of CD34^+^ and CD34^−^ cells in a murine hind-limb ischemia model. To elucidate further the role of hematopoietic cells in EPC differentiation, we isolated different populations of hematopoietic cells. T lymphocytes (CD3^+^) markedly accelerated the early EPC status of CD34^+^ cells, while macrophages (CD11b^+^) or megakaryocytes (CD41^+^) specifically promoted large EPC colonies.

**Conclusion:**

Our results suggest that specific populations of hematopoietic cells play a role in the EPC differentiation of CD34^+^ cells, a finding that may aid in the development of a novel cell therapy strategy to overcome the quantitative and qualitative limitations of EPC therapy.

## Introduction

Endothelial progenitor cells (EPCs) contribute to the neovascularization in response to ischemic signals, and have been reported as potential biomarkers of cardiovascular disease [1]–[6]. Although EPC therapy has been studied as a new strategy in regenerative medicine, various methods of culture of different EPC populations with distinct properties have been explored in the study and management of ischemic diseases [7]–[10]. Several studies examining ischemic sites have reported that different types of transplanted EPCs promoted a robust vascular regeneration and were therapeutic in ischemic vascular disease [11]–[15]. Although many investigators speculate that EPCs are responsible for the modest effects observed in ischemic disease, little is known about the actual mechanism of EPC differentiation.

Research on human EPCs has been ambiguous, mainly owing to the lack of a precise definition of EPC and proper EPC assay. Recently, EPCs have been qualified and quantified by cell-surface markers including CD34, CD133, and vascular endothelial growth factor receptor-2 (VEGFR-2) [16], or conventional EPC culture methods including EPC culture assay, CFU-EC colony assay, and endothelial colony forming cell (ECFC) assay from several primary blood samples such as peripheral blood, bone marrow, or umbilical cord [10]. These assays have contributed to the evaluation of the developmental and vasculogenic properties of EPCs, but have been disputed regarding the quality, quantity, and identification of primary circulating EPCs [17]. Our group has developed a new clonogenic assay system, which is a modification of a conventional methylcellulose assay used for identification of stem and progenitor cells [18]. This novel EPC-colony forming assay (EPC-CFA) allows an assessment of the fundamental and proper qualification and quantification of EPCs [18]–[20]. The EPC-CFA discriminates between two types of EPC colony-forming units (EPC-CFUs), such as small EPC-CFUs, which present proliferative capabilities, and large EPC-CFUs, which present vasculogenic properties.

Putative endothelial cell progenitors or angioblasts were isolated from human peripheral blood based on their expression of CD34 [1], a molecule expressed by hematopoietic stem cells, hematopoietic progenitor cells, and microvascular endothelial cells. In several studies, the use of CD34^+^ cell populations in vascular regeneration therapy has been performed in various preclinical and clinical trials [21]–[27]. CD34^+^ cells markedly accelerated the rate of restoration of blood flow to the ischemic limb, while CD34^−^ cells produced no effect [21], [28]. Nevertheless, the significance of CD34^−^ cell populations has been highlighted. The subsets of both CD34^+^ and CD34^−^ cells are capable of long-term hematopoietic repopulation [29]–[31]. CD34^−^/CD133^+^ EPC subpopulations are precursors of classical CD34^+^/CD133^+^ EPCs, but are functionally more potent with respect to homing and vascular regeneration [12]. CD34^−^/CD45^+^ bone marrow stromal cells have cardioprotective activity against ischemia/reperfusion injury [32]. CD34^−^/Lin^−^/CD45^−^/CD133^−^ cells are able to generate functional endothelial cells that contribute to neovascularization [33]. However, there are few reports that indicate a relation between CD34^+^ and CD34^−^ cells in the regulation of EPC differentiation and function.

Considering these findings, we focused on cross talk between CD34^+^ cells and CD34^−^ cells and determined their importance in EPC differentiation using the EPC-CFA, which is a novel method to assess the EPC colony-forming potential of stem cells. We also evaluated the functional properties of EPC-CFUs, such as adhesion, tube formation, and migration, and their functional recovery in a murine hind-limb ischemia model on the basis of the presence of CD34^−^ populations. Furthermore, we investigated which population of CD34^−^ cells affects the differentiation of CD34^+^ cells into EPCs. This study clarified whether specific cross talk between CD34^+^ cells and hematopoietic cells regulates CD34^+^ cell differentiation into EPCs.

## Materials and Methods

### Animals

Experiments were performed on male 8-wk-old Balb/C nude mice (Biogenomics, Seoul, Korea, http://www.orient.co.kr) maintained under a 12-hour light/dark cycle and in accordance with the regulations of Pusan National University. The protocols were approved by the Institutional Animal Care and Use Committee of Pusan National University School of Medicine, based on the Guide for the Care and Use of Laboratory Animals.

### Ethical statement

After obtaining informed, written consent, human umbilical cord blood was collected from healthy volunteers according to a protocol approved by the Ethics Review Board of the Hospital of the Pusan National University of YangSan, Korea. The Institutional Animal Care and Use Committee of Pusan National University, YangSan, Korea approved all surgical interventions and post-operative animal care. The approved protocol number is IACUC090017.

### Isolation of CD34^+^ cells

Human umbilical cord blood (HUCB) was supplied by the Pusan National University Hospital. CD34^+^ cells were isolated from HUCB as reported previously [20]. Briefly, total mononuclear cells (MNCs) were isolated by Ficoll (GE Healthcare, Buckinghamshire, U.K.) density gradient centrifugation of the cord blood. The CD34^+^ cells were separated from MNCs using magnetic-activated cell sorting (MACS) (CD34^+^ Microbead Kit; Miltenyi Biotec, Bergisch Gladbach, Germany) according to the manufacturer’s instructions, to a final purity of more than 98%.

### EPC colony-forming assay

Human CD34^+^, CD34^−^, or both CD34^+^ and CD34^−^ cells isolated from HUCB were cultured in methylcellulose-containing medium, H4236 (StemCell Technologies, Vancouver, Canada), supplemented with 20 ng/mL stem cell-derived factor (Kirin, Tokyo, Japan), 50 ng/mL vascular endothelial growth factor (VEGF; R&D Systems, Minneapolis, MN, USA), 20 ng/mL interleukin (IL)-3 (Kirin), 50 ng/mL basic fibroblast growth factor (bFGF; Wako, Osaka, Japan), 50 ng/mL epidermal growth factor (EGF; Wako), 50 ng/mL insulin-like growth factor (IGF)-1 (Wako), 2 U/mL heparin (Ajinomoto, Tokyo, Japan), and 10% fetal bovine serum (FBS; Life Technologies, Carlsbad, CA) on a 35-mm dish (Thermo SCIENTIFIC, Rockford, IL) for 8 d. The cell density of each sample was 5×10^2^ cells per dish or was adjusted depending on the assay. The EPCs were identified as small EPC-CFUs or large EPC-CFUs by visual inspection using a light microscope (OLYMPUS, Tokyo, Japan) under 40x magnification. Small EPC-CFUs were composed of round adhesive cells, and large EPC-CFUs were composed of spindle-shaped cells. Nonattached cells were isolated as small EPCs by washing with PBS (WELGENE, Daegu, Korea), while attached cells were harvested as large EPCs by treatment with 5 mM EDTA (Sigma-Aldrich, St. Louis, MO) in PBS (5 mmol/L) for 5 min at 37°C.

### EPC-CFU staining

After 8 days in culture, the EPC-CFU cultures were treated with 0.4 µg/mL 1,1′-dioctadecyl-3,3,3′,3-tetramethyl-indocarbocyanine perchlorate-labeled acLDL (acLDL-DiI; Biomedical Technologies Inc., Stoughton, MA) for 1 h and fixed by application of 1 mL of 2% paraformaldehyde (PFA) (Affymetrix, Santa Clara, CA) for 1 h at room temperature. After washing with methylcellulose-PBS, the cultures were reacted with fluorescein isothiocyanate (FITC)-conjugated lectin from *Ulex europaeus* (UEA-I; Sigma-Aldrich) for 1 h at room temperature. The cultures were washed with PBS and then imaged using a confocal fluorescence microscope (Olympus).

### Coculture analysis

Coculture analysis was performed in 12-well Millicell Cell Culture Plates (0.4 µm pore size; Millipore, Billerica, MA, USA) using Stem Span serum-free medium (StemCell Technologies), supplemented with 50 ng/mL VEGF, 20 ng/mL IL-6 (R&D Systems), 100 ng/mL SCF (Kirin), 20 ng/mL thrombopoietin (TPO; Wako), 100 ng/mL Flt-3 ligand (Wako), and 1% penicillin-streptomycin (WELGENE). Human CD34^+^ cells isolated from HUCB were seeded in the lower compartment of the transwell, and the transwell membrane inserts were seeded with human CD34^−^ cells isolated from HUCB or left unseeded. To measure the EPC-CFU potential of CD34^+^ cells after coculture, the transwell inserts were removed after 7 d. The expansion of CD34^+^ cell was determined by cell counting.

### Adhesive assay

Ninety-six-well culture plates were coated with human fibronectin (100 µg/mL; Life Technologies). EPC-CFUs (1×10^4^ cells per well) were allowed to attach in endothelial basal medium 2 (EBM-2; Lonza, Walkersville, MD) supplemented with 5% fetal bovine serum (FBS; Lonza), human vascular endothelial growth factor (hVEGF; Lonza), human basic fibroblast growth factor (bFGF; Lonza), human epidermal growth factor (hEGF; Lonza), human insulin-like growth factor 1 (hIGF-1; Lonza), ascorbic acid (Lonza), and GA-1000 (Lonza) (EGM-2; Lonza) for 20 min at 37°C, and the nonadherent cells were then aspirated. The adherent cells were measured after 4 h of incubation with detection reagents using the Cell Counting Kit-8 (DOJINDO, Kumamoto, Japan) with detection at 490 nm using a microplate reader (Tecan, Männedorf, Switzerland).

### Tube formation assay

Human umbilical vein endothelial cells (HUVECs) were purchased from Lonza and were cultured in EGM-2 following the supplier’s instructions. Each group–media only, CD34^+^ cells, mixed CD34^+^ and CD34^−^ cells, CD34^+^ cell culture medium, or mixed CD34^+^ and CD34^−^ cell culture medium–was mixed with a sample of HUVECs (with a ratio of cell fractions of each group to HUVEC of 1×10^3^ to 1.2×10^4^ cells in a 50-µl volume of 2% FBS in EBM-2) was seeded onto a 96-well plate (Thermo SCIENTIFIC) coated with Matrigel (10 mg/mL, BD Biosciences, San Diego, CA) and further incubated for 8 h. After incubation, the number of branching points was counted using one picture per well at 40x magnification by light microscopy.

### Migration assay

The migration capacity of HUVECs in response to CD34^−^ cells was measured using the Boyden chamber assay (Cell Biolabs, San Diego, CA). Cells (3×10^5^) were placed in the upper part of a Boyden chamber. The chamber was placed in a 24-well culture dish (Thermo SCIENTIFIC) containing EBM-2, EGM-2, or EGM-2 containing CD34^−^ cells for 24 h at 37°C. For quantification, cells migrating to the lower chamber were counted in 5 random microscopic fields using a hemocytometer (Marienfeld Superior, Lauda-Königshofen, Germany). Results are expressed as the number of migrated cells per 300,000 cells added to the Boyden chamber.

### Cell transplantation in a hind-limb ischemia murine model

All procedures were performed in accordance with the policies of the Pusan National University of Korea institutional animal care and use committee. In the hind-limb ischemia murine model, ischemia was induced by ligating the proximal femoral artery and boundary vessels of 8-wk-old Balb/C nude mice. No later than 6 h after operation, CD34^+^ or CD34^+^/CD34^−^ cell-derived EPC-CFUs in PBS were transplanted via intramuscular injection into the ischemic thigh area (5×10^5^ cells/100 µL PBS per mouse), and blood perfusion using laser Doppler perfusion imaging (LDPI; Moor Instruments, Wilmington, DE) and capillary density by immunohistochemistry for rabbit polyclonal mouse-specific anti-platelet-endothelial cell adhesion molecule 1 (anti-PECAM-1; CD31, Santa Cruz Biotechnology, Santa Cruz, CA) were assessed. For cell tracking analysis, transplanted human EPC-CFUs were labeled with anti-human nuclei monoclonal antibody (anti-HNA; Millipore).

### Flow cytometry analysis

Several subpopulations were isolated from HUCB CD34^−^ cells to assess their EPC-CFU capacity in coculture with CD34^+^ cells. Each subpopulation (of macrophages, T cells, B cells, megakaryocytes, and monocytes) was isolated using a sterile live cell flow cytometry sorter (BD FACSAria; BD Biosciences) using labeled anti-human CD11b, CD3, CD19, CD41, and CD14 antibodies (BD Biosciences). To characterize EPC-CFUs, small and large EPC-CFUs were labeled with anti-human CD11b, CD14, CD45, and CD144 antibodies (BD Biosciences), and analyzed.

### Statistical analysis

The statistical comparison of the 2 groups was performed using Student’s *t*-test. The results were analyzed using the Statview 5.0 software package (Abacus Concepts, Inc., CA). Scheffé’s test was performed for multiple comparisons between each group after ANOVA. All data, which were obtained from at least 3 independent experiments, were expressed as means ± standard deviation.

## Results

### Differential status of EPC development in response to HUCB CD34^+^ and CD34^−^ cells

**Figure 1:**
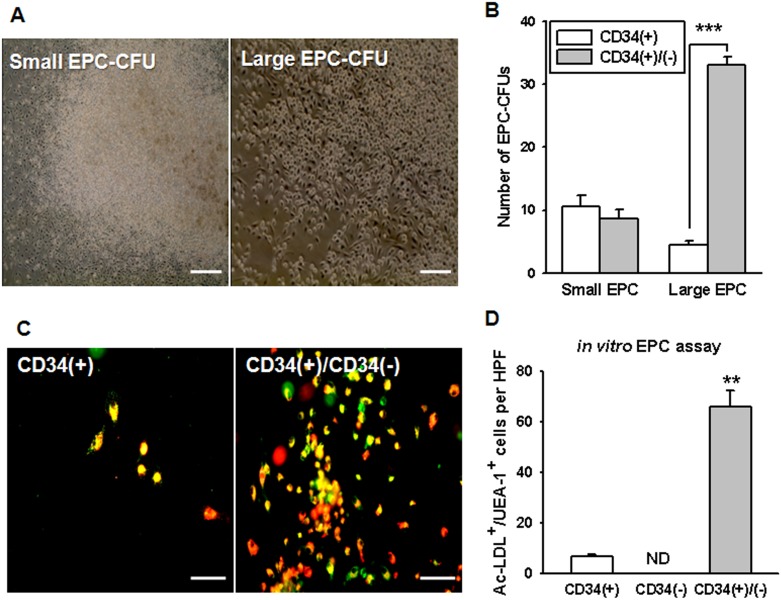
EPC-CFA profiles of HUCB CD34^+^ cells and mixed CD34^+^ and CD34^−^ cells. (A) Morphology of small EPC-CFUs and large EPC-CFUs derived from HUCB CD34^+^ cells (Scale bar = 20 µm). (B) EPC differentiation and expansion potentials were estimated by quantification of the 2 types of EPC-CFU clusters in CD34**^+^** cell-derived EPC-CFUs and mixed CD34**^+^** and CD34^−^ cell-derived EPC-CFUs. The results are shown as means ± SEM (***p<0.001 *vs*. CD34**^+^** cell-derived EPC-CFUs). (C) Endothelial characteristics of CD34^+^ cell-, CD34^−^ cell-, and mixed CD34**^+^** and CD34^−^ cell-derived EPC-CFUs were assessed for Ac-LDL uptake (red) and *Ulex europaeus* agglutinin I (UEA-1)-conjugated FITC binding (green) (Scale bar = 100 µm). (D) Standard quantification of endothelial characteristics of EPC-CFUs was performed by counting the number of double-stained cells. The results are shown as means ± SEM (**p<0.01 *vs*. CD34**^+^** cell-derived EPC-CFUs). CD34^−^ cells could not be differentiated into either type of EPC-CFU clusters.

To determine the status of EPC development of CD34^+^, CD34^−^, or mixed CD34^+^ and CD34^−^ cells isolated from HUCB, we first established a novel EPC colony-forming assay (EPC-CFA) as described previously (2,000 CD34^+^ or CD34^−^ cells per 35 mm well) [18]. EPC colony-forming units (EPC-CFUs) formed 2 types of clusters, small EPC-CFUs and large EPC-CFUs. Small EPC-CFUs contained mainly small and round adhesive cells, whereas large EPC-CFUs contained spindle-shaped cells (Figure 1A). FACS analysis was performed to characterize each EPC-CFU. Small and large EPC-CFUs expressed surface markers of monocytic lineages: CD11, CD14, and CD45. Large EPC-CFUs expressed the endothelial marker, CD144, but small EPC-CFUs did not (Figure S1). In the EPC-CFA, we determined the EPC colony-forming potential of CD34^+^, CD34^−^, or mixed CD34^+^ and CD34^−^ cells (Figure 1B). The number of small EPC-CFUs was not significantly different when comparing the CD34^+^ cells and mixed CD34^+^/CD34^−^ cells, but the number of large EPC-CFUs was significantly increased in mixed CD34^+^/CD34^−^ cells compared with cultures of CD34^+^ cells alone. Both types of EPC-CFUs were positive for acetylated low-density lipoprotein (Ac-LDL) uptake and for *Ulex europaeus* agglutinin I (UEA-I) binding, confirming the endothelial cell (EC) lineage (Figure 1C). Quantitative analysis of 5 random visual fields under a fluorescence microscope revealed that the number of double-positive cells was significantly increased in mixed CD34^+^/CD34^−^ cells compared with CD34^+^ cells (Figure 1D). CD34^−^ cells did not form either type of EPC-CFU in the EPC-CFA. These results reveal that CD34^−^ cells can substantially increase the *in vitro* EPC differentiation of CD34^+^ cells.

### The EPC colony-forming potential of CD34^+^ cells in the presence of CD34^−^ cells

**Figure 2:**
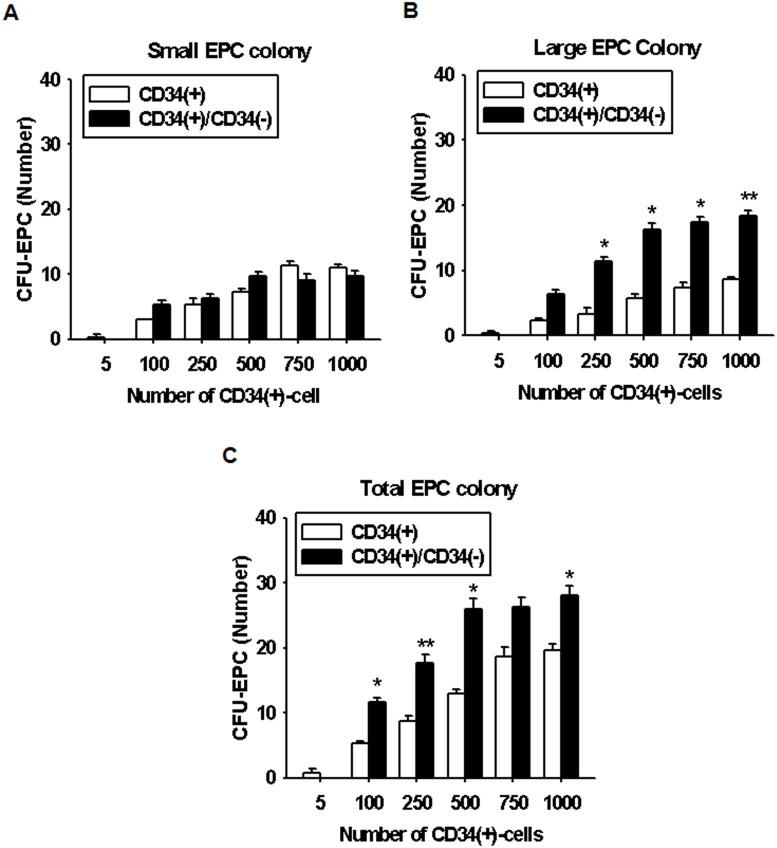
Assessment of EPC differentiation and expansion potentials according to the number of HUCB CD34^+^ cells. EPC differentiation and the expansion potential were investigated after coculture of varying densities of CD34^+^ cells (5, 100, 250, 500, 750, and 1000 cells per 35-mm dish) with CD34^−^ cells (1000 cells per 35-mm dish). Standard quantification of EPC-CFUs was performed by counting the number of small, large, and total EPC-CFUs. (A) The number of the small EPC-CFUs was not significantly different among the groups. (B and C) Large EPC-CFUs and total EPC-CFUs were significantly increased in the CD34^+^-CD34^−^ cell coculture compared to that in CD34^+^ cell culture alone condition. The results are shown as means ± SEM (*p<0.05 and **p<0.01 *vs*. CD34**^+^** cell-derived EPC-CFUs).

CD34^+^ cells grown in the presence of CD34^−^ cells have been shown to have a higher rate of differentiation into EPCs (Figure 1). To investigate the optimal ratio of CD34^+^ and CD34^−^ cells for EPC differentiation, we initially examined whether the number of CD34^+^ cells and the presence of CD34^−^ cells can affect EPC-CFU formation. Different amounts of CD34^+^ cells, 5, 100, 250, 500, 750, and 1000 cells per dish, with or without 1000 CD34^−^ cells, were plated. The frequencies of small EPC colonies were not significantly different between CD34^+^-only cell culture and the CD34^+^/CD34^−^ cell coculture groups (Figure 2A), whereas the frequencies of large and total EPC colonies were significantly increased in coculture of CD34^+^ cells with CD34^−^ cells compared with CD34^+^-only cell culture (Figure 2B and C). The frequencies of EPC-CFUs gradually increased with increasing number of CD34^+^ cells; however, in the groups where more than 500 CD34^+^ cells were cultured with CD34^−^ cells, the frequencies of EPC-CFUs were not significantly increased compared to that in the group of 500 CD34^+^ cells with CD34^−^ cells. These findings suggest that the optimal number of CD34^+^ cells on a 35 mm dish is 500 CD34^+^ cells per dish.

**Figure 3:**
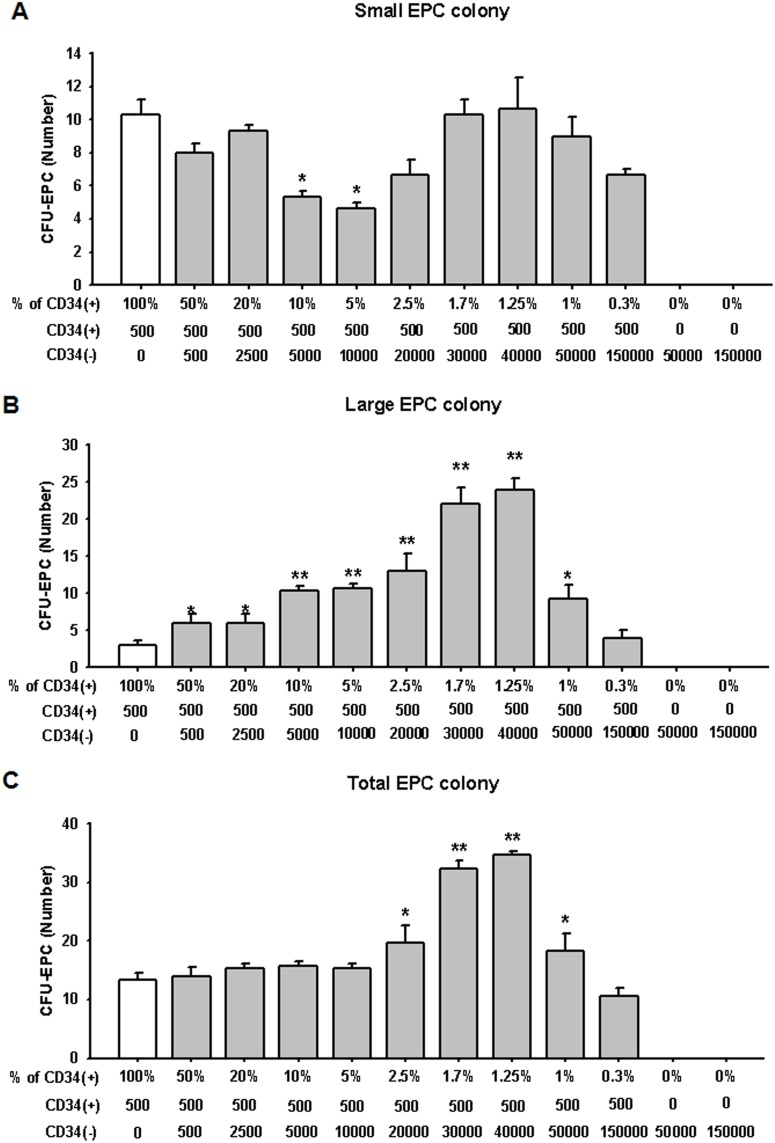
Optimal proportion CD34^+^ cells to CD34^−^ cells for effective EPC differentiation. The optimal proportion of CD34^+^ cells to CD34^−^ cells was estimated by coculture of CD34^+^ cells (500 cells per 35-mm dish) with various densities of CD34^−^ cells (100, 50, 20, 10, 5, 2.5, 1.7, 1.25, 1, 0.3, and 0% of CD34^+^ cells). (A) Small EPC colonies were not significantly increased in all groups. (B and C) Large EPC-CFUs and total EPC-CFUs were significantly increased in coculture of CD34^+^ and CD34^−^ cells compared with CD34^+^ cell culture only in several groups. The results are shown as means ± SEM (*p<0.05 and **p<0.01 *vs*. CD34**^+^** cell-derived EPC-CFUs).

We then investigated the optimal ratio between CD34^+^ and CD34^−^ cells for differentiation of EPCs. After culturing CD34^+^ cells (500 cells per dish) with various ratios of CD34^−^ cells–50, 20, 10, 5, 2.5, 1.7, 1.25, 1, 0.3, and 0% of CD34^+^ cells–we assessed 2 types of EPC colony-forming potentials by measuring the frequency of EPC-CFUs. No correlation was observed between small EPC colony-forming capacity and an increase in the CD34^−^ cell number (Figure 3A). On the other hand, coculture of 1.25% CD34^+^ cells with CD34^−^ cells showed the highest large and total EPC colony-forming capacity among the groups (Figures 3B and C). These results indicate that an optimal ratio between CD34^+^ and CD34^−^ cells is important in promoting EPC differentiation.

### Assessment of the functional capacity of EPC-CFUs in response to CD34^−^ cells

**Figure 4:**
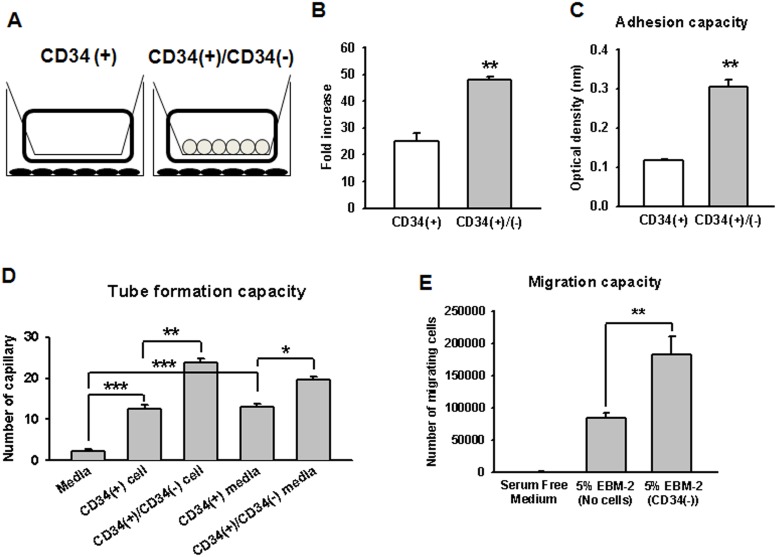
Assessment of the function of EPC-CFUs in response to CD34^−^ cells. (A) A schematic diagram to evaluate the effect of CD34^−^ cells on the expansion of CD34^+^ cells using a transwell culture system. (B) Standard quantification of the expansion of CD34^+^ cells was performed by calculating the number of expanded CD34^+^ cells (lower chamber) using transwell cultures with or without CD34^−^ cells (upper chamber). The results are shown as means ± SEM (**p<0.01 *vs.* transwell culture of CD34^+^ cells without CD34^−^ cells). (C) Adhesion capacity was estimated by culture on human fibronectin-coated well plates. Standard quantification of adhesion cells was analyzed by the Cell Counting Kit-8 with detection at 490 nm using a microplate reader. The results are shown as means ± SEM (**p<0.01 *vs.* CD34^+^ cell-derived EPC-CFUs). (D) Tube formation capacity was assessed by tube formation assay of HUVECs cocultured with EC culture medium, CD34^+^ cells, mixed CD34^+^ and CD34^−^ cells, conditioned medium from cultured CD34^+^ cells, and conditioned medium from cocultured CD34^+^ and CD34^−^ cells in Matrigel. The results are shown as means ± SEM (*p<0.05, **p<0.01, and ***p<0.001 *vs.* each group). (E) Migration was determined by the Boyden chamber assay with HUVECs treated with serum- and growth factor-free EC medium, complete EC medium, and complete EC medium containing CD34^−^ cells for 24 h. Migrated cells were counted in 5 random microscopic fields in the lower chamber by a hemocytometer with Cell Counting Kit-8 with detection at 490 nm using a microplate reader. The results are shown as means ± SEM (**p<0.01 *vs.* the complete EC medium-treated group).

To determine the effect of CD34^−^ cells on CD34^+^ cells, we examined the functional capacity of EPC-CFUs, including proliferation and the capacity for adhesion, tube formation, and migration. We performed a transwell coculture assay to characterize the paracrine effect of CD34^−^ cells on the proliferation of CD34^+^ cells (Figure 4A). The proliferation ratio was approximately 2-fold higher when CD34^+^ cells were cocultured with CD34^−^ cells, than in CD34^+^-only cell culture (Figure 4B). To evaluate the adhesion capacity, we developed a cell adhesion assay. As expected, coculture of CD34^+^ cells with CD34^−^ cells (1.25% CD34^+^ cells with CD34^−^ cells) increased the adhesive capacity compared to that induced by CD34^+^ cell culture alone (Figure 4C). Next, we performed a tube formation assay on HUVECs with CD34^+^ or CD34^−^ cells because CD34^+^ cells are immature and early-phase EPCs. To determine the effect of CD34^−^ cells on EC-derived tube formation, we cocultured HUVECs with CD34^+^ cells or CD34^+^ and CD34^−^ cells (1.25% CD34^+^ cells with CD34^−^ cells), or incubated them with conditioned medium from CD34^+^ cell culture, conditioned medium from CD34^+^ and CD34^−^ cell coculture (1.25% CD34^+^ cells with CD34^−^ cells), or medium alone (as a control), on Matrigel. All subset groups could substantially promote tube formation by HUVECs compared with the control group. Interestingly, EC-derived tube formation was the highest when cells were incubated with the coculture of CD34^+^ and CD34^−^ cells, or the conditioned medium from such a coculture (Figure 4D), suggesting the importance of not only cell-to-cell interaction but also paracrine factors from CD34^−^ cells in EC-derived tube formation. In addition, CD34^−^ cells resulted in a better migratory capacity of ECs than serum- and cytokine-free medium or complete EC medium in the Boyden chamber (Figure 4E). These findings indicate that CD34^−^ cells might play a pivotal role in the functional regulation of EPC-CFUs and ECs through cell-to-cell interaction and paracrine effects.

### Contribution of CD34^−^ cells to the functional enhancement of transplanted EPC-CFUs in postnatal neovascularization

**Figure 5:**
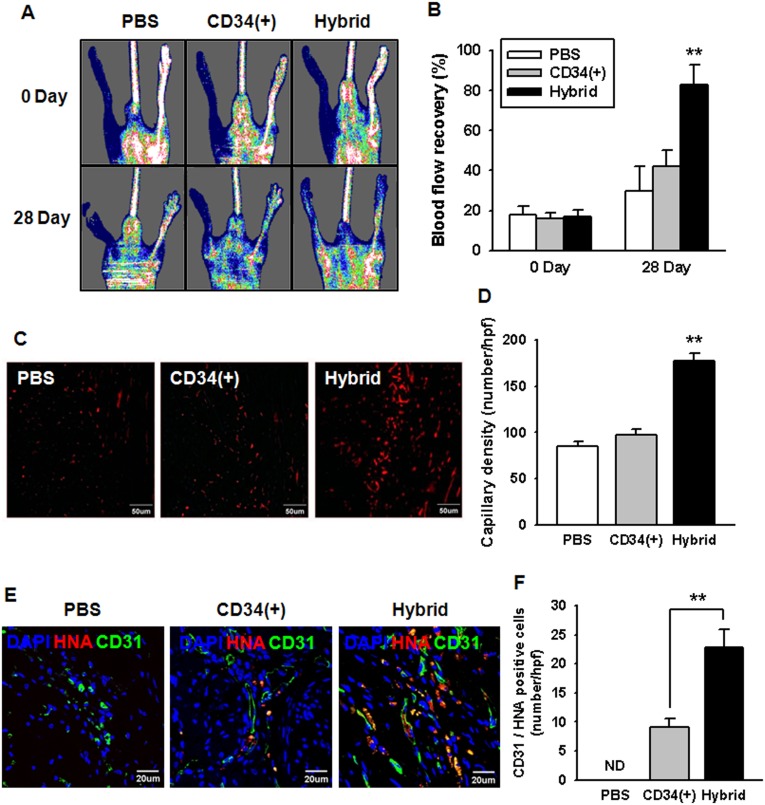
Assessment of the functional recovery in murine hind-limb ischemia. (A) Laser Doppler perfusion imaging (LDPI) analysis of murine hind-limb ischemia transplanted with PBS, CD34^+^ cell-derived large EPC-CFUs (CD34(+)), and mixed CD34^+^ and CD34^−^ cell (1.25% CD34^+^ cells with CD34^−^ cells)-derived large EPC-CFUs (Hybrid) on days 0 and 28 post surgery. (B) Blood flow recovery ratio obtained by dividing the blood flow of the ischemic (left) limb by that of the nonischemic (right) limb. LDPI was measured on days 0 and 28 post operation. The results are shown as means ± SEM (**p<0.01 *vs.* the PBS-transplanted group). (C) At postoperative day 28, ischemic limb tissues were analyzed for the formation of capillary by staining for anti-CD31 (red) antibodies. (D) Standard quantification of the capillary density was evaluated by counting CD31^+^ cells per high-power field (hpf). The results are shown as means ± SEM (**p<0.01 *vs.* the PBS-transplanted group). (E) At postoperative day 28, ischemic limb tissues were analyzed for the engraftment of large EPC-CFUs into CD31-positive vessels by staining for anti-HNA (red) and anti-CD31 (green) antibodies. (F) Standard quantification of the engraftment of large EPC-CFUs into CD31-positive vessels was evaluated by counting CD31 and HNA-positive cells per hpf. The results are shown as means ± SEM (**p<0.01 *vs.* the CD34^+^ cell-derived large EPC-CFUs transplanted group).

A previous study revealed that large CFU-EPCs play an important role in the restoration of ischemic diseases [19]. We used a murine hind-limb ischemia model to evaluate the postnatal neovascularization of transplanted large EPC-CFUs. We transplanted CD34^+^ cell-derived large EPC-CFUs and mixed CD34^+^ and CD34^−^ cell (1.25% CD34^+^ cells with CD34^−^ cells)-derived large EPC-CFUs into the ischemic sites, and controls were treated with PBS. To assess the recovery of blood flow, the blood flow was measured by laser Doppler perfusion imaging (LDPI) at postoperative day 28 (Figure 5A). The recovery of blood flow was significantly improved when mice received mixed CD34^+^ and CD34^−^ cell-derived large EPC-CFUs, compared with that in animals in any other group (Figure 5B). Moreover, immunohistochemical staining for CD31 confirmed that capillary formation was established in the ischemic tissue at postoperative day 28 (Figure 5C). Immunohistochemical staining of CD31 was distinctly increased in vessels following transplantation of mixed CD34^+^ and CD34^−^ cell-derived large EPC-CFUs compared with any other treatment (Figure 5D). To explore the engraftment of transplanted large EPC-CFUs, immunohistochemical staining of CD31 and HNA, which specifically labels human cells, was established in the ischemic tissue at postoperative day 28 (Figure 5E). CD31 and HNA-positive cells were detected in the ischemic limbs, suggesting the incorporation of transplanted large EPC-CFUs into CD31-positive vessels. The number of HNA-positive cells in CD31-positive vessels was increased in mixed CD34^+^ and CD34^−^ cell-derived large EPC-CFUs compared with any other treatment (Figure 5F). These data suggest that CD34^−^ cells may contribute to the functional enhancement of transplanted EPC-CFUs for neovascularization.

### Identification of the cell population integral to differentiation of EPCs

**Figure 6:**
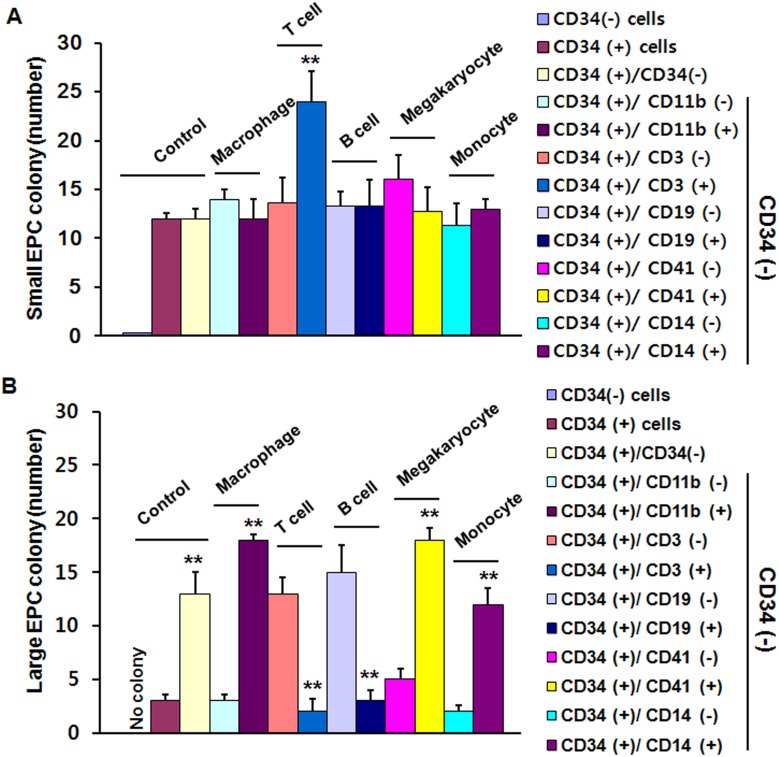
Identification of hematopoietic cell populations that regulate EPC development from HUCB CD34^+^ cells. Standard quantification of small EPC colonies (A) and large EPC colonies (B) was performed by counting the number of each type of colony cluster after EPC-CFA of HUCB CD34^+^ cells cocultured with hematopoetic cells (HUCB CD34^−^) which consist of various cells such as macrophages (CD11b^+^ and CD11b^−^ cells), T cells (CD3^+^ and CD3^−^ cells), B cells (CD19^+^ and CD19^−^ cells), megakaryocytes (CD41^+^ and CD41^−^ cells), and monocytes (CD14^+^ and CD14^−^ cells) on CD34^−^ cells that were isolated using FACS analysis for specific markers from CD34^−^ cells. Control represents each number of colony clusters of HUCB CD34^−^ cells, CD34^+^ cells, or coculture of CD34^+^ and CD34^−^ cells. The other bars, macrophage, T cell, B cell, megakaryocte, and monocyte represents each number of colony clusters of HUCB CD34^+^ with CD 11b^−^ or with CD 11b^+^, with CD3^−^ or with CD3^+^, with CD19^−^ or with CD19^+^, with CD41^−^ or with CD41^+^, and with CD14^−^ or with CD14^+^, respectively. The results are shown as means ± SEM (**p<0.01 *vs.* culture of CD34^+^ only or coculture of CD34^+^ and various negative cells such as CD11b^−^, CD3^−^, CD19^−^, CD41^−^, or CD14^−^ cells, respectively).

To identify the essential cell population in the differentiation of EPCs in CD34^−^ cell fractions, we performed the EPC-CFA by culturing CD34^+^ cells with various fundamental hematopoietic cells including macrophages, T cells, B cells, megakaryocytes, and monocytes. To isolate various hematopoietic cell populations from CD34^−^ cells, we first examined the expression of CD3 (a T cell marker), CD11b (a macrophage marker), CD14 (a monocyte marker), CD19 (a B cell marker), and CD41 (a megakaryocyte marker) on CD34^−^ cells by FACS analysis. Then, to assess the EPC colony-forming capacity of CD34^+^ cells, we established an EPC-CFA for CD34^+^ cells with cell populations positive or negative for CD3, CD11b, CD14, CD19, and CD41 on CD34^−^ cells. Small EPC-CFUs were more significantly increased when CD34^+^ cells were cocultured with CD3^+^ cells than any other cell type (Figure 6A), whereas large EPC-CFUs were more significantly increased when CD34^+^ cells were cocultured with CD11b^+^, CD41^+^, and CD14^+^ cells compared with the other cell types (Figure 6B). These results show that T cells may have an essential role in the differentiation of small EPC-CFUs, while macrophages, monocytes, and megakaryocytes may be important in the differentiation of large EPC-CFUs.

## Discussion

Although several studies in the last decade have reported the pivotal role of EPCs in ischemic diseases after their initial isolation [1], the definitive delineation of EPCs remains a challenge due to the lack of a clear hierarchy of differentiation and a defined isolation protocol. Thus, evidence of the efficacy of EPCs in peripheral arterial disease is limited, and the impact of isolated EPCs remains undetermined [27], [34]–[39]. The EPC-CFA system allows us to assess the fate and vasculogenic potential of circulating EPCs from primary cells, based on a numerical evaluation of their hierarchical adhesive clonogenicity.

In this study, we performed EPC-CFAs [18] to evaluate the status of EPC development in response to specific interactions with CD34^−^ cells, and found that hematopoietic cells support the development of stem cell-derived EPCs. The EPC-CFA was designed to identify EPC-CFUs from CD34^+^ cells, an EPC-enriched population. Two types of EPC-CFUs derived from CD34^+^ cells could be identified: “small EPC-CFUs,” which are composed of small round-shaped cells with proliferative capacity and are defined as primitive EPCs, and “large EPC-CFUs,” which are large spindle-shaped cells with vasculogenic potential and are defined as definitive EPCs [3], [17]. Our previous studies revealed that each EPC-CFU expressed surface markers of the endothelial and monocytic lineages [18], and that CD34^−^ cells increased the expression of endothelial lineage markers, such as VEGFR2, Tie2, and CXCR4 [20]. These results correspond well with our findings in the present study (Figure S1), indicating that EPC-CFUs are immature EPCs as pro-angiogenic cells and that CD34^−^ cells facilitate a higher expression level of EPC functional markers.

We investigated specific interactions between CD34^+^ and CD34^−^ cells by evaluating EPC-CFU differentiation of CD34^+^ cells. Contrary to CD34^+^ cell culture alone, the large EPC colony-forming capacity was markedly enhanced following coculture of CD34^+^ cells with CD34^−^ cells, although the small EPC colony-forming capacity was not significantly enhanced. The proliferation capacity, adhesion capacity, incorporation into tubes formed by EC-like cells, and migratory capacity of the large EPCs also dramatically increased in EPC-CFUs derived from coculture of CD34^+^ cells with CD34^−^ cells compared with those derived from CD34^+^ cell culture alone. This finding suggests that CD34^−^ cells may play a pivotal role as supporting cells in EPC differentiation and regulate the function of EPCs through cell-to-cell interactions and paracrine actions during *ex vivo* culture. In particular, CD34^−^ cells influence the large EPC colony-forming capacity, providing new insights into efficient cell culture strategies to generate functional EPCs.

**Figure 7:**
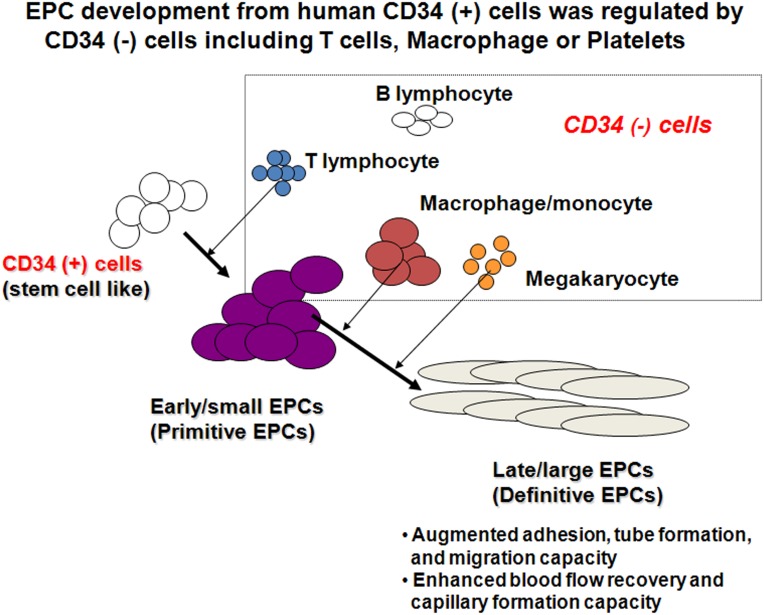
Schematic model of the status of EPC development through specific cross talk with key hematopoietic cells isolated from CD34^−^ cells. EPC-CFA identifies the EPC differentiation hierarchy, early/small EPCs (primitive EPCs), and late/large EPCs (definitive EPCs) by the morphological and functional hallmarks. In EPC-CFA, CD34^−^ cells, in particular T lymphocytes, macrophages, monocytes, and megakaryocytes, play a pivotal role in EPC development. T lymphocytes activate the differentiation CD34^+^ cells into primitive EPCs, which are round-shaped and proliferative. Macrophages, monocytes, and megakaryocytes accelerate the differentiation CD34^+^ cells and/or primitive EPCs into definitive EPCs, augmenting the adhesion, tube formation, and migration capacity, and enhancing the functional recovery, blood flow recovery, and capillary formation capacity, *in vivo*.

Based on the above evidence that CD34^−^ cells affect the EPC differentiation status, we investigated which hematopoietic cells originating from CD34^−^ cell fractions are the key regulators of EPC development and differentiation from CD34^+^ cells. It was previously reported that a T cell subset (human CD3^+^/CD31^+^/CXCR4^+^ T cells) is critical in the formation of EPC colonies [40]. A previous report using EPC-CFA also showed that development of early EPCs is augmented by an increase in CD14, a monocyte marker, during EPC differentiation [18]. Circulating CD34^+^/KDR^+^ cells are generated from circulating multipotent CD34^+^ cells at platelet-rich sites [41]. In this study, we found that T cells increased the formation of small EPC colonies, which are immature and proliferative EPCs (primitive EPCs), whereas macrophages, monocytes, and megakaryocytes increased the formation of large EPC colonies, which are more mature and functional EPCs, through cell-to-cell interactions and paracrine effects (Figure 7).

We then evaluated the contribution of CD34^−^ cells to the vasculogenic potential of EPC-CFUs of CD34^+^ cells in a murine hind-limb model, and found the recovery of blood flow and capillary formation capacity to be significantly enhanced with administration of mixed CD34^+^ and CD34^−^ cell-derived EPC-CFUs, suggesting that CD34^−^ cells contribute to the production of proliferative and functionally enhanced EPC-CFUs from CD34^+^ cells.

To our knowledge, this study is the first to show that hematopoietic cells–which are CD34^−^ cells, including macrophages, monocytes, T cells, B cells, and megakaryocytes–contribute to the formation of total EPC colonies composed of small and large EPC-CFUs and play a role in the development of the endothelial lineage. Additionally, the optimal proportion of CD34^+^ cells to CD34^−^ cells in EPC-CFA, which produces proliferative and functional definitive EPCs, was found to be as follows: coculture of 1.25% CD34^+^ cells with CD34^−^ cells.

## Conclusions

The findings of this study highlight EPC development in response to specific interactions with CD34^−^ cells, including hematopoietic cells, through a qualitative and quantitative analysis of EPCs using a clonogenic culture system, the EPC-CFA. Our results suggest that coculture of an optimal proportion of CD34^+^ cells to CD34^−^ cells may assist in the development of a novel cell therapy that surmounts the restrictions of CD34^+^ cell yield in high-risk patients and produces effective EPCs. This study provides valuable information to overcome the limitations of cell therapy and may help in the development of more efficient approaches than the ones currently used.

## Supporting Information

Figure S1 **Flow cytometric analysis of cell-surface markers in EPC-CFUs.** CD34^+^ cell-derived small EPC-CFUs (CD34(+) small EPC-CFUs), CD34^+^ cell-derived large EPC-CFUs (CD34(+) large EPC-CFUs), mixed CD34^+^ and CD34^−^ cell-derived small EPC-CFUs (Hybrid small EPC-CFUs), and mixed CD34^+^ and CD34^−^ cell-derived large EPC-CFUs (Hybrid large EPC-CFUs) were labeled with anti-CD11b, CD14, CD45 and CD45 antibodies and evaluated by flow cytometric analysis. Histograms indicate the ratios of CD11b, CD14, CD45 and CD45 expression. Representative images from four independent experiments are shown.
(TIF)
