# Supplementary figures and images for: Cross Talk with Hematopoietic Cells Regulates the Endothelial Progenitor Cell Differentiation of CD34 Positive Cells
Source: PLoS One. 2014 Aug 28;9(8):e106310. doi: 10.1371/journal.pone.0106310 (PMC4148437; doi:10.1371/journal.pone.0106310)

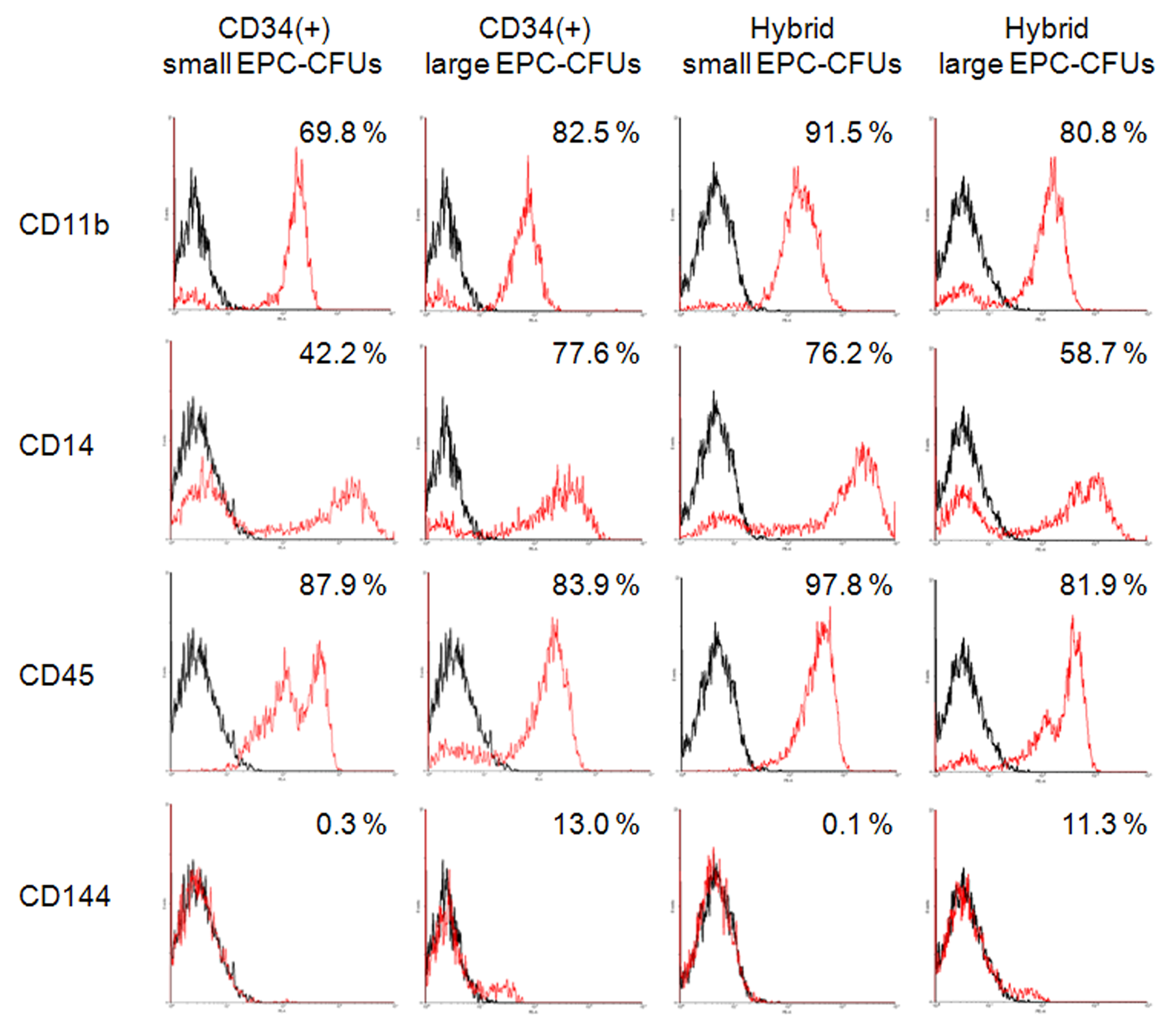

Supplement: Figure S1 — Flow cytometric analysis of cell-surface markers in EPC-CFUs. CD34+ cell-derived small EPC-CFUs (CD34(+) small EPC-CFUs), CD34+ cell-derived large EPC-CFUs (CD34(+) large EPC-CFUs), mixed CD34+ and CD34− cell-derived small EPC-CFUs (Hybrid small EPC-CFUs), and mixed CD34+ and CD34− cell-derived large EPC-CFUs (Hybrid large EPC-CFUs) were labeled with anti-CD11b, CD14, CD45 and CD45 antibodies and evaluated by flow cytometric analysis. Histograms indicate the ratios of CD11b, CD14, CD45 and CD45 expression. Representative images from four independent experiments are shown. (TIF) [file pone.0106310.s001.tif]
